# Supplementary material for: Chronic spindle assembly checkpoint activation causes myelosuppression and gastrointestinal atrophy
Source: EMBO Rep. 2024 May 28;25(6):15. doi: 10.1038/s44319-024-00160-3 (PMC11169569; doi:10.1038/s44319-024-00160-3)
Supplement: Supplementary file 1 — Appendix [file 44319_2024_160_MOESM1_ESM.pdf]

Appendix table of contents

Title	Page
Appendix Figure S1: Systemic MAD2 overexpression perturbs leukocyte homeostasis.	2-3
Appendix Figure S2: Mad2 transgenic blood cells are outcompeted over time in vivo.	4
Appendix Figure S3: MAD2 overexpression does not perturb leukocyte development.	5
Appendix Figure S4: BMF expression in vitro and in vivo after MAD2 transgene induction.	6

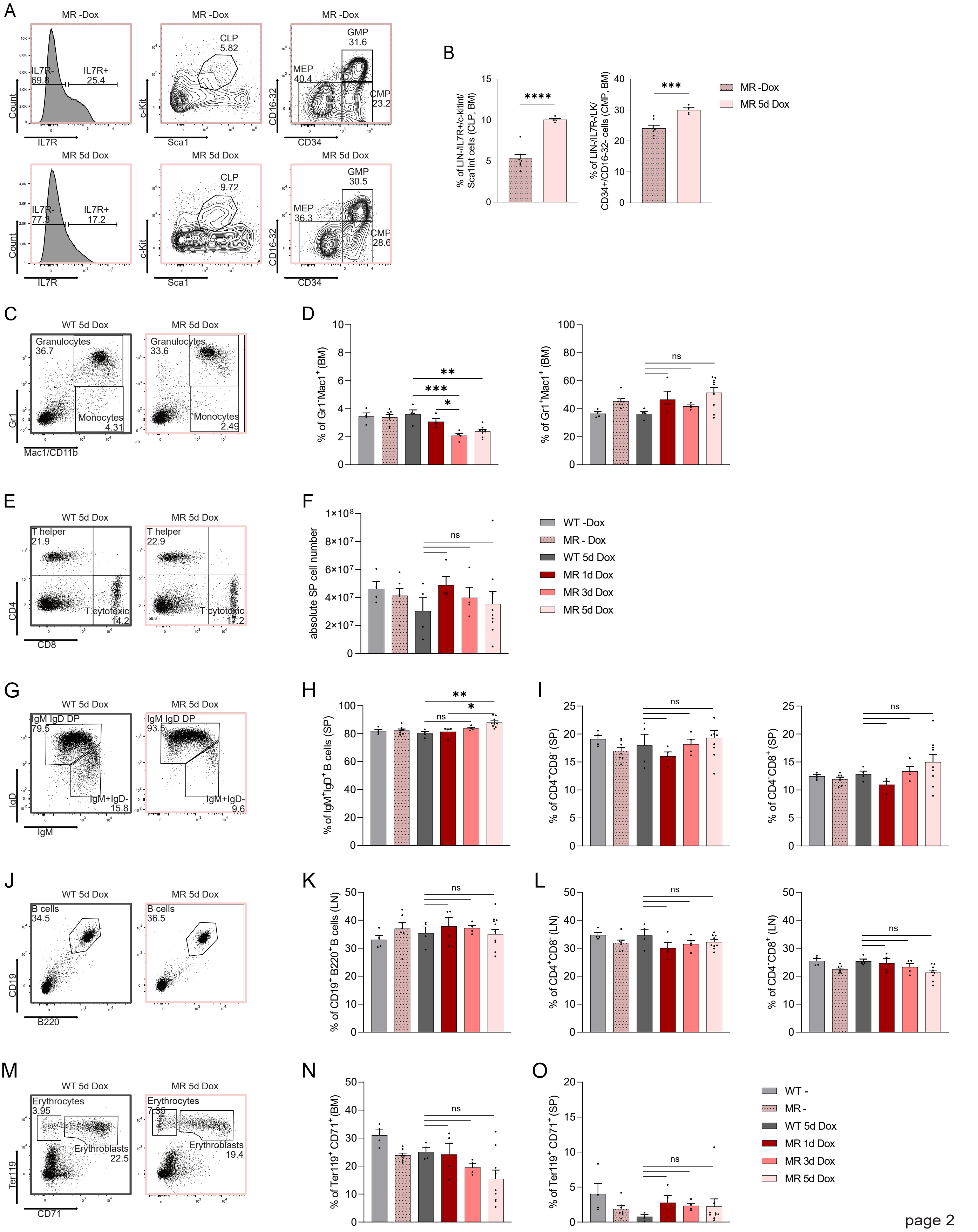
A
MR -Dox
IL7R-
69.8
IL7R+
25.4
Count
IL7R
CLP
5.82
c-Kit
Sca1
GMP
31.6
MEP
40.4
CMP
23.2
CD16-32
CD34
MR 5d Dox
IL7R-
77.3
IL7R+
17.2
CLP
9.72
GMP
30.5
MEP
36.3
CMP
28.6
B
% of LIN-/IL7R+/c-kitint/ Sca1int cells (CLP, BM)
****
% of LIN-/IL7R-/LK/ CD34+/CD16-32- cells (CMP, BM)
***
MR -Dox
MR 5d Dox
C
WT 5d Dox
MR 5d Dox
Granulocytes
36.7
Monocytes
4.31
Granulocytes
33.6
Monocytes
2.49
Gr1
Mac1/CD11b
D
% of Gr1-Mac1+ (BM)
% of Gr1+Mac1+ (BM)
ns
E
T helper
21.9
T cytotoxic
14.2
T helper
22.9
T cytotoxic
17.2
CD4
CD8
F
absolute SP cell number
WT -Dox
MR - Dox
WT 5d Dox
MR 1d Dox
MR 3d Dox
MR 5d Dox
G
IgM IgD DP
79.5
IgM+IgD-
15.8
IgM IgD DP
93.5
IgM+IgD-
9.6
IgD
IgM
H
% of IgM+IgD+ B cells (SP)
I
% of CD4+CD8- (SP)
% of CD4-CD8+ (SP)
J
B cells
34.5
B cells
36.5
CD19
B220
K
% of CD19+ B220+ B cells (LN)
L
% of CD4+CD8- (LN)
% of CD4-CD8+ (LN)
M
Erythrocytes
3.95
Erythroblasts
22.5
Erythrocytes
7.35
Erythroblasts
19.4
Ter119
CD71
N
% of Ter119+ CD71+ (BM)
O
% of Ter119+ CD71+ (SP)
WT -
MR -
WT 5d Dox
MR 1d Dox
MR 3d Dox
MR 5d Dox

Appendix Figure S1: Systemic MAD2 overexpression perturbs leukocyte homeostasis.

A Gating strategy used in flow cytometric analyses of bone marrow (BM) of MR mice kept on regular diet (MR-Dox) or 5 days on Dox food (MR 5d Dox). Common lymphoid progenitors (CLP, $Lin^{-}IL7R^{+}ckit^{int}Sca1^{int}$), common myeloid progenitors (CMP, $Lin^{-}IL7R^{+}ckit^{+}Sca1^{-}CD34^{+}CD16/32^{-}$).

B Quantification of data in A. MR-Dox (n=6), MR 5d Dox (n=4).

C Gating strategy used in flow cytometric analyses of BM stained for granulocytes ($Gr1^{+}CD11b^{+}$) and monocyte/macrophages ($Gr1^{-}CD11b^{+}$).

D Quantification of data shown in C.

E Gating strategy used in flow cytometric analyses of spleen (SP) stained for T helper ($CD4^{+}CD8^{-}$) and T cytotoxic ($CD4^{-}CD8^{+}$) cells.

F SP cellularity. (G) Gating strategy used in flow cytometric analyses for $IgM^{+}IgD^{+}$ cells of total B cells ($CD19^{+}B220^{+}$).

H Quantification of data shown in G.

I Quantification of data shown in E.

J Gating strategy used in flow cytometric analyses lymph nodes (LN) stained for B cell markers ($CD19^{+}B220^{+}$).

K Quantification of data shown in J.

L LN stained for T helper cell ($CD4^{+}CD8^{-}$) and cytotoxic T cell markers ($CD4^{-}CD8^{+}$).

M Gating strategy used in flow cytometric analyses of bone marrow (BM) stained for erythroblasts ($Terr119^{+}CD71^{+}$).

N Quantification of data shown in M.

O Quantification of spleen (SP) stained for erythroblasts ($Terr119^{+}CD71^{+}$).

C-O: WT-Dox (n=3), MR-(n=7), WT 5d Dox (n=3), MR 1d Dox (n=3), MR 3d Dox (n=4), MR 5d Dox (n=9).

Data information: B-O Data shown as mean ± SEM. B Unpaired t-test, Welchs correction. D-O One-Way ANOVA, Tukey's multiple comparisons. ns: not significant, *P≤0.05, **P≤0.01, ***P≤0.001, ****P≤0.0001.

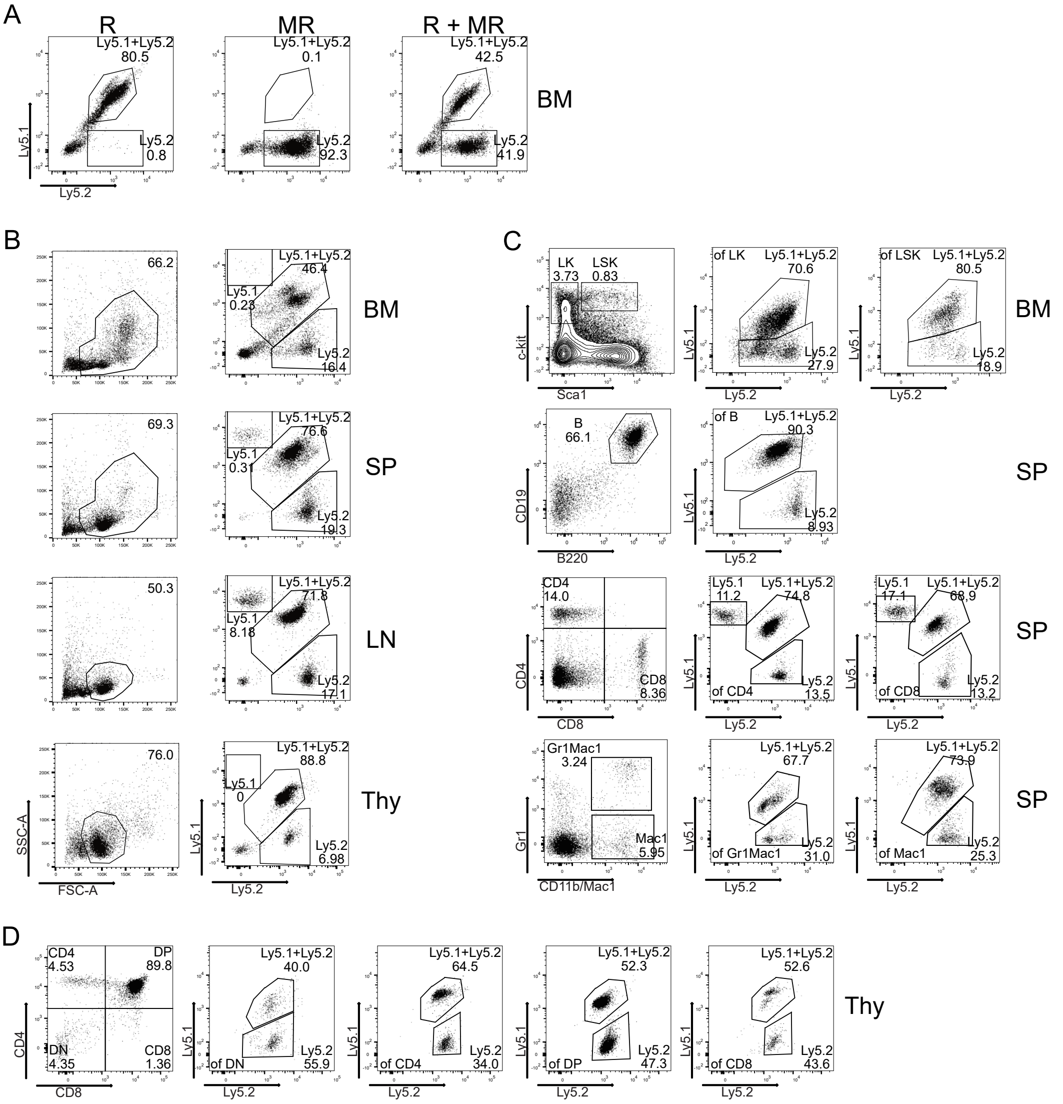

Appendix Figure S2: Mad2 transgenic blood cells are outcompeted over time in vivo. Gating strategy of flow cytometric data shown in Figure 2.

A Ly5.1/2 and Ly5.2 expression of donor BM cells used for 50:50 reconstitution experiments.

B Assessment of Ly5.1 and Ly5.2 expression levels in total haematopoietic organs (BM: bone marrow, SP: spleen, LN: lymph node, Thy: Thymus) 12 weeks after reconstitution and housing animals on Dox-containing food.

C Cell type identification and corresponding Ly5.1, Ly5.1/2 or Ly5.2 expression in BM and SP.

D Thymocyte subset analysis using anti-CD4 and anti-CD8 antibodies in combination with Ly5.1 and Ly5.2 expression markers.

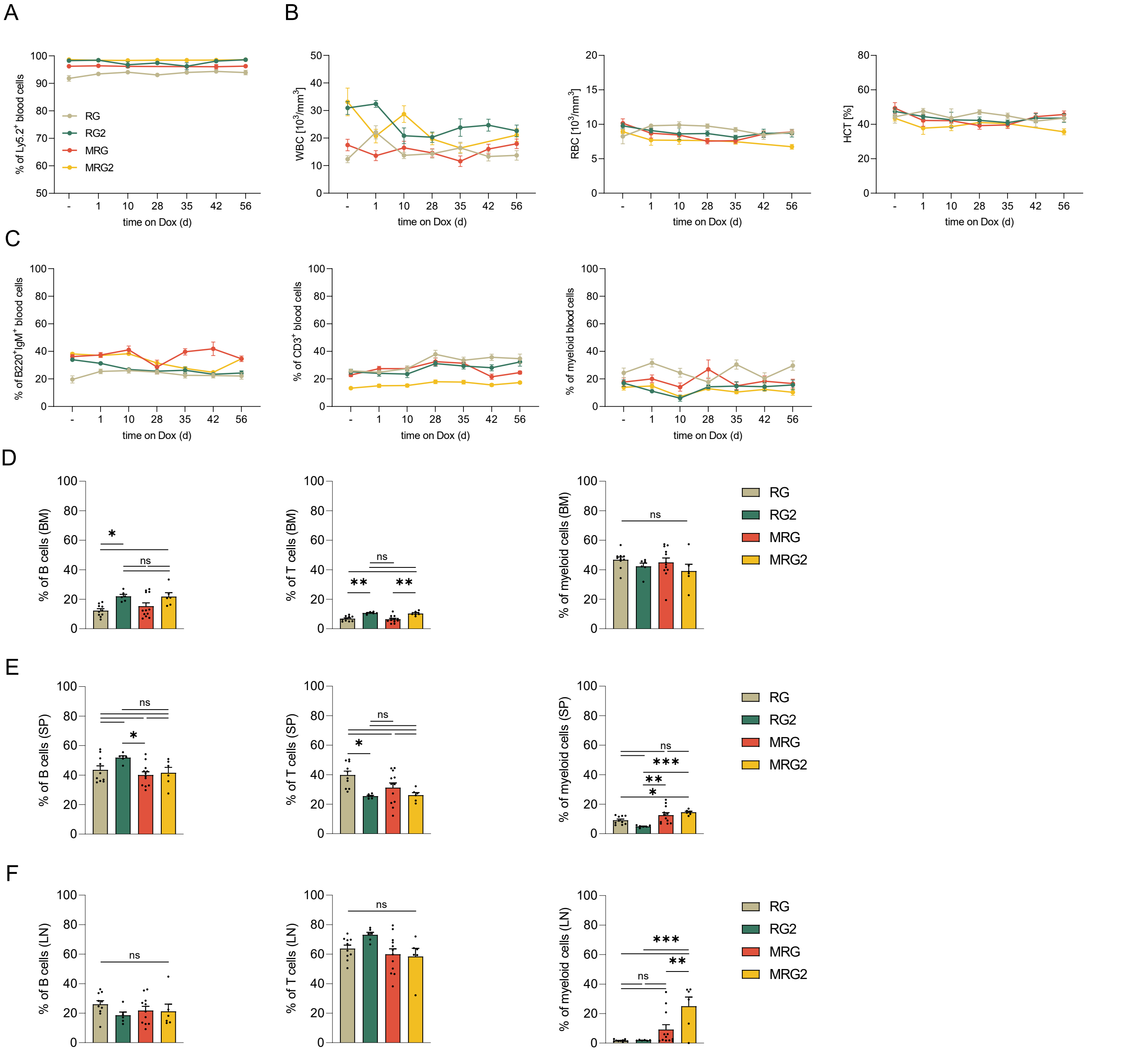

Appendix Figure S3: MAD2 overexpression does not perturb leukocyte development.

A Percentage of donor-derived Ly5.2^{+} cells in the blood of reconstituted animals before (-) and after diet change to Dox-containing food.

B White (WBC), red blood cell count (RBC) and haematocrit (HCT) of reconstituted animals.

C Composition of peripheral blood was assessed by flow cytometry using marker for B (B220^{+}IgM^{+}), T (CD3^{+}), and myeloid cells (Gr1$^{+/-}$CD11b^{+}).

D,E,F (D) BM (E) SP and (F) LN (lymph node) of reconstituted animals were analysed by flow cytometry after 10 weeks on Dox-containing food. Cells were stained with antibodies to identify total B cells (CD19^{+}B220^{+}), T (CD4^{+}&CD8^{+}) and myeloid cells (Gr1$^{+/-}$CD11b^{+}).

A-F RG (n=8-10), RG2 (n=6), MRG (n=11-12), MRG2 (n=6).

Data information: A-F Data shown as mean ± SEM. D-F One-Way ANOVA, Tukey's multiple comparisons. ns not significant, *P≤0.05, **P≤0.01, ***P≤0.001.

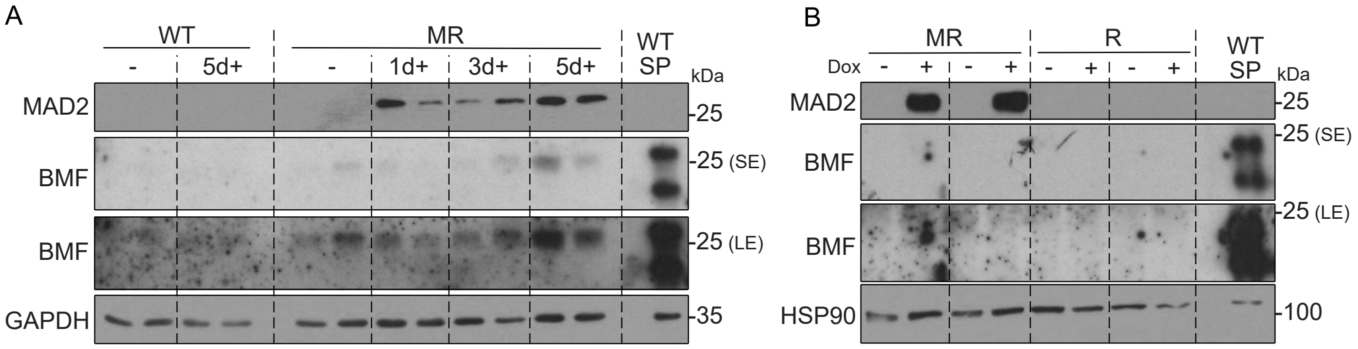

Appendix Figure S4: BMF expression *in vitro* and *in vivo* after MAD2 transgene induction.

A Colonic extracts of WT and MAD2 transgenic (MR) mice on Dox food (1d+, 3d+, 5d+) or normal diet (-) for indicated time points were subjected to immunoblotting using the indicated antibodies. Lysates from 2 mice are shown. Spleen (SP) of one WT mouse was loaded as positive control for BMF expression.

B MAD2 transgenic (MR) and rtTA (R) HoxB8-PF progenitor cells were treated with or without 1µg/ml Doxycycline for 24h and subjected to immunoblot analysis with indicated antibodies. Spleen (SP) of one WT mouse was used as positive control for BMF expression.
