# Supplementary material for: Chronic spindle assembly checkpoint activation causes myelosuppression and gastrointestinal atrophy
Source: EMBO Rep. 2024 May 28;25(6):15. doi: 10.1038/s44319-024-00160-3 (PMC11169569; doi:10.1038/s44319-024-00160-3)
Supplement: Supplementary file 7 — Expanded View Figures [file 44319_2024_160_MOESM7_ESM.pdf]

Expanded View Figures

Figure EV1. Characterization of cellular responses to MAD2 overexpression.

HoxB8 cell lines were generated from the bone marrow of mice of the indicated genotype treated with (+) or without (-) Doxycycline for the indicated times. (**A**) HoxB8-PF cells were harvested after 48 h of transgene induction with different Doxycycline concentrations for immunoblot analysis using the indicated antibodies. (**B**) HoxB8-PF cells were treated with 1 µg/ml Doxycycline or left untreated for the indicated times and harvested for immunoblot analysis using the indicated antibodies. (**C**, **D**) Gating strategy used in flow cytometric analyses of cells shown in Fig. 1C,D. (**E**) HoxB8-PN cells were incubated with and without Doxycycline (1 µg/ml). Cell death was followed in Incucyte live-cell microscopy by PI uptake; pictures were taken every 2 h. MR ($n = 3$; 2 biological replicates), MR2 ($n = 5$; 3 biological and 1–2 technical replicates); data shown as mean ± SEM. (**F**, **G**) (**F**) HoxB8-PN cells were analyzed for GFP reporter expression before and during Doxycycline (1 µg/ml) treatment in PI-negative cells by FACS (MRG $n = 4$, MRG2 $n = 1$) and on (**G**) protein level (*: indicates residual HA-MAD2 signal after re-probing the membrane with anti-GFP antibody). (**H**) HoxB8-PF cells were analyzed for dead cells (Annexin V/DAPI) and GFP reporter expression before and during Doxycycline (1 µg/ml) treatment by FACS. MRG ($n = 4$, 2 biological and 2 technical replicates), RG ($n = 6$, 3 biological and 2 technical replicates); data shown as mean ± SEM. (**I**) HoxB8-PFs were sorted for the expression of GFP (negative, low and high) after 24 h of Doxycycline (1 µg/ml) treatment and transgene induction and subjected for immunoblot analysis using indicated antibodies. Data information: (**E**, **F**, **H**) Data shown as mean ± SEM. (**H**) Two-way ANOVA, Sidak's multiple comparisons. ns not significant, $^*P \leq 0.05$,

 © The Author(s)

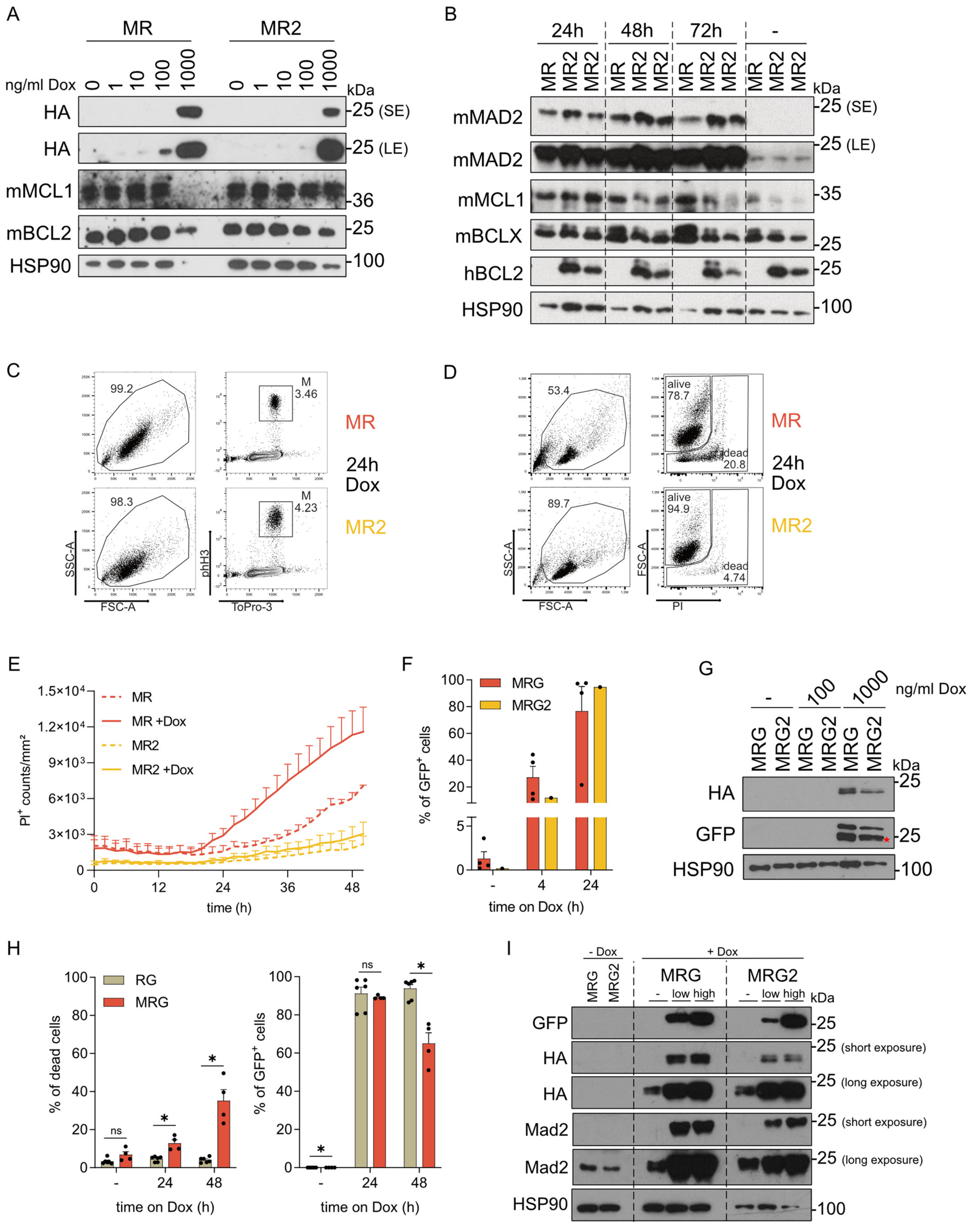
A
MR
MR2
ng/ml Dox
0 1 10 100 1000
kDa
HA
-25 (SE)
-25 (LE)
mMCL1
-36
mBCL2
-25
HSP90
-100
B
24h 48h 72h -
MR MR2 MR2
mMAD2
mMCL1
-35
mBCLX
hBCL2
C
99.2
98.3
M 3.46
M 4.23
MR
24h Dox
MR2
SSC-A
FSC-A
phH3
ToPro-3
D
53.4
89.7
alive 78.7
dead 20.8
alive 94.9
dead 4.74
PI
FSC-A
E
MR
MR +Dox
MR2
MR2 +Dox
PI+ counts/mm²
time (h)
F
MRG
MRG2
% of GFP+ cells
time on Dox (h)
G
ng/ml Dox
MRG MRG2
GFP
H
RG
MRG
% of dead cells
ns
time on Dox (h)
I
- Dox
+ Dox
low high
(short exposure)
(long exposure)
Mad2

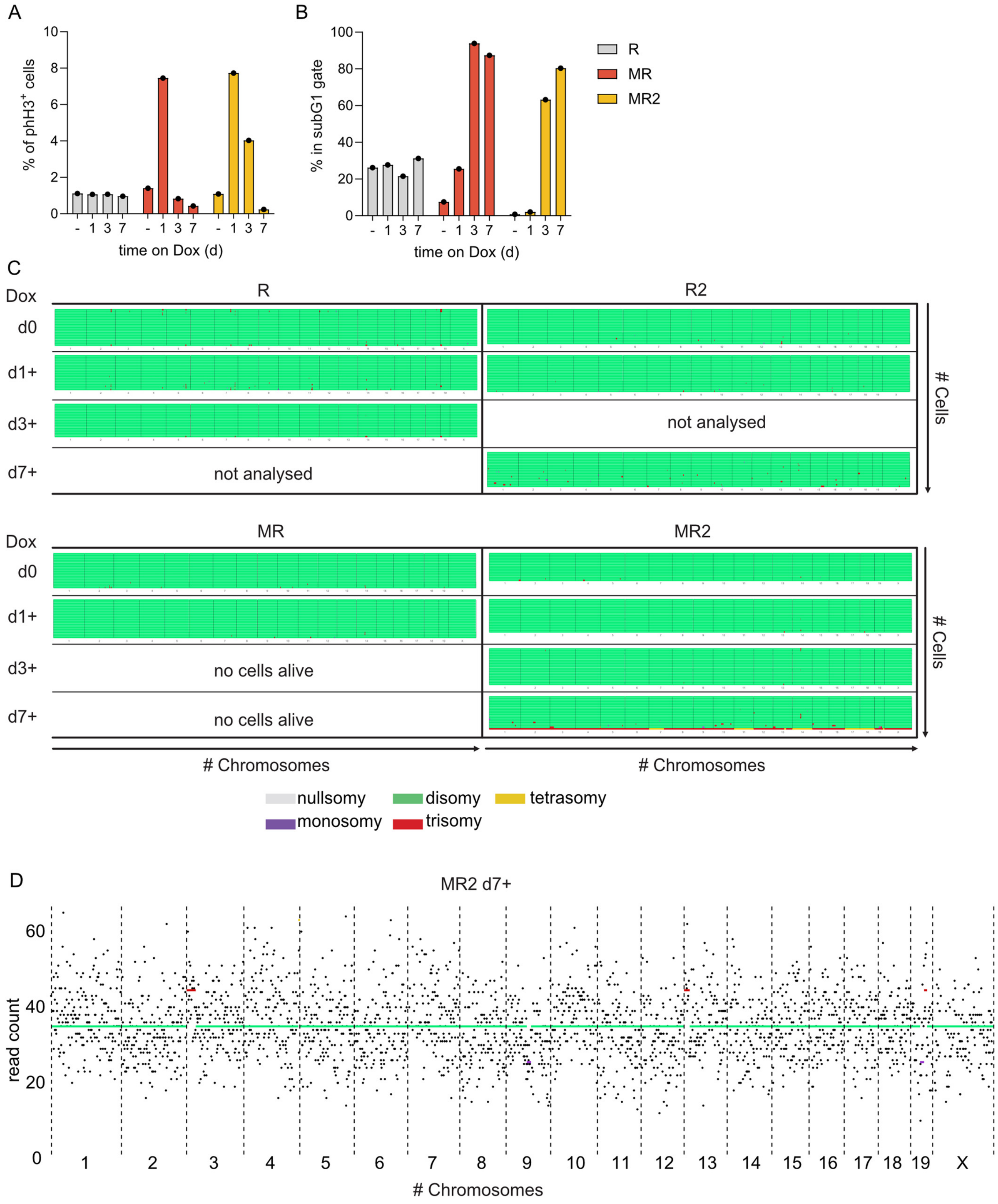

Figure EV2. HoxB8 cells do not show CIN upon MAD2 overexpression.

(**A**, **B**) Cells used for scWGS were fixed and analyzed for quantification of (**A**) mitotic cells (phH3+) and (**B**) viability (sub-G1 staining) by FACS ($n = 1$). (**C**) scWGS analyses of cells in (**A**, **B**) over time. (**D**) Representative read count of MR2 cell line 7 days on Doxycycline with euploid karyotype.

 © The Author(s)

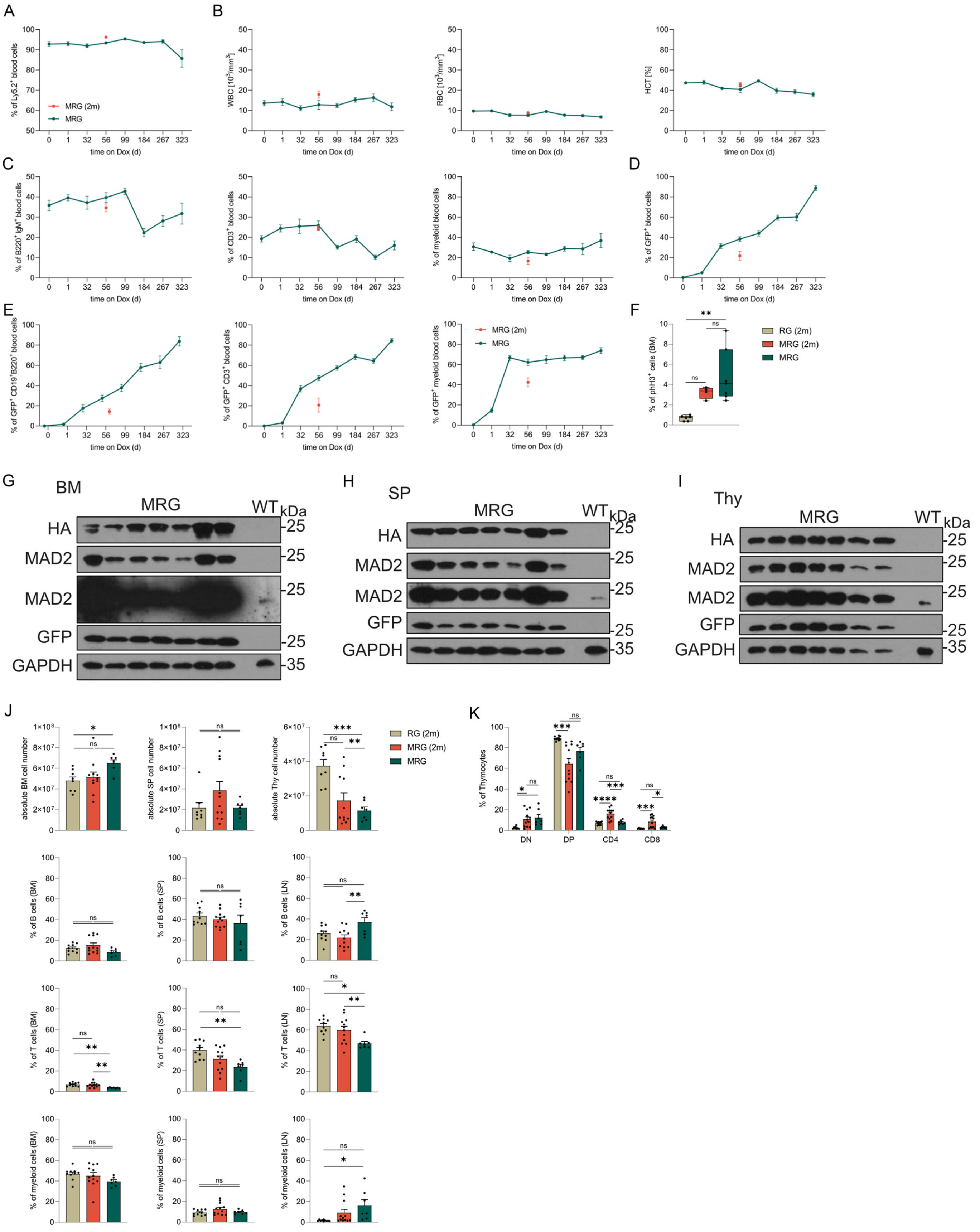
A
B
C
D
E
F
G
H
I
J
K
% of Ly5.2+ blood cells
WBC [10³/mm³]
RBC [10⁶/mm³]
HCT [%]
% of B220+ IgM+ blood cells
% of CD3+ blood cells
% of myeloid blood cells
% of GFP+ blood cells
% of GFP+ CD19+B220+ blood cells
% of GFP+ CD3+ blood cells
% of GFP+ myeloid blood cells
% of phH3+ cells (BM)
time on Dox (d)
0 1 32 56 99 184 267 323
MRG (2m)
MRG
RG (2m)
BM
SP
Thy
MRG
WT
kDa
HA
MAD2
GFP
GAPDH
25
35
absolute BM cell number
absolute SP cell number
absolute Thy cell number
% of Thymocytes
DN
DP
CD4
CD8
% of B cells (BM)
% of B cells (SP)
% of B cells (LN)
% of T cells (BM)
% of T cells (SP)
% of T cells (LN)
% of myeloid cells (BM)
% of myeloid cells (SP)
% of myeloid cells (LN)
ns

Figure EV3. Long-term MAD2 overexpression does not trigger blood cancer.

$Ly5.1^{+}$ animals were reconstituted with BM derived from $Ly5.2^{+}$ MRG donors, set on Dox food after 12 weeks of reconstitution and monitored over 12 month (MRG n = 7). Data of reconstituted animals from our first cohort, analyzed after 2 months (2 m) on Dox food (shown in Fig. 4 and Appendix Figure S3), is displayed again for reference purposes here, RG (2 m) $n = 6$–10 and MRG (2 m) $n = 4$–12. (**A**) Peripheral blood was stained to quantify the percentage of $Ly5.2^{+}$ cells. (**B**) White (WBC), red blood cell (RBC) counts and hematocrit (HCT) were determined using a ScilVet abc blood cell analyser. (**C**) Analysis of mature B cells ($B220^{+}IgM^{+}$), T cells ($CD3^{+}$) and myeloid cells ($Gr1^{+/-}CD11b^{+}$) in peripheral blood over time. (**D**, **E**) GFP expression in total blood and individual blood cell types. (**F**) Bone marrow (BM) was stained using antibodies specific for phH3 to identify mitotic cells. (**G**–**I**) (**G**) BM, (**H**) spleen (SP) and (**I**) thymus (Thy) of reconstituted mice were analyzed for MAD2 transgene expression after 12 month on Dox-containing food by western blot (WT: non-reconstituted WT mouse). (**J**) Cellularity of BM, SP and Thy as well as leukocyte content were analyzed and compared to that seen in mice 2 month after reconstitution. Cell surface marker-specific antibodies were used to identify total B cells ($CD19^{+}B220^{+}$), T ($CD4^{+}$&$CD8^{+}$) and myeloid cells ($Gr1^{+/-}CD11b^{+}$). (**K**) Thymocytes were stained with antibodies specific for CD4 and CD8 to identify DN ($CD4^{-}CD8^{-}$), DP ($CD4^{+}CD8^{+}$), T helper ($CD4^{+}CD8^{-}$) and T cytotoxic ($CD4^{-}CD8^{+}$) thymocyte subsets. Data information: (**A**–**E**, **J**, **K**) Data shown as mean ± SEM. (**F**) Data shown as Min to Max with median and IQR of: RG (2 m) 0.5, MRG (2 m) 0.78, MRG 4.64. (**F**, **J**) One-way ANOVA, Tukey's multiple comparisons. (**K**) One-way ANOVA within each subset (DN, DP, CD4, CD8). ns not significant, $^{*}P \leq 0.05$, $^{**}P \leq 0.01$, $^{***}P \leq 0.001$, $^{****}P \leq 0.0001$.

© The Author(s)

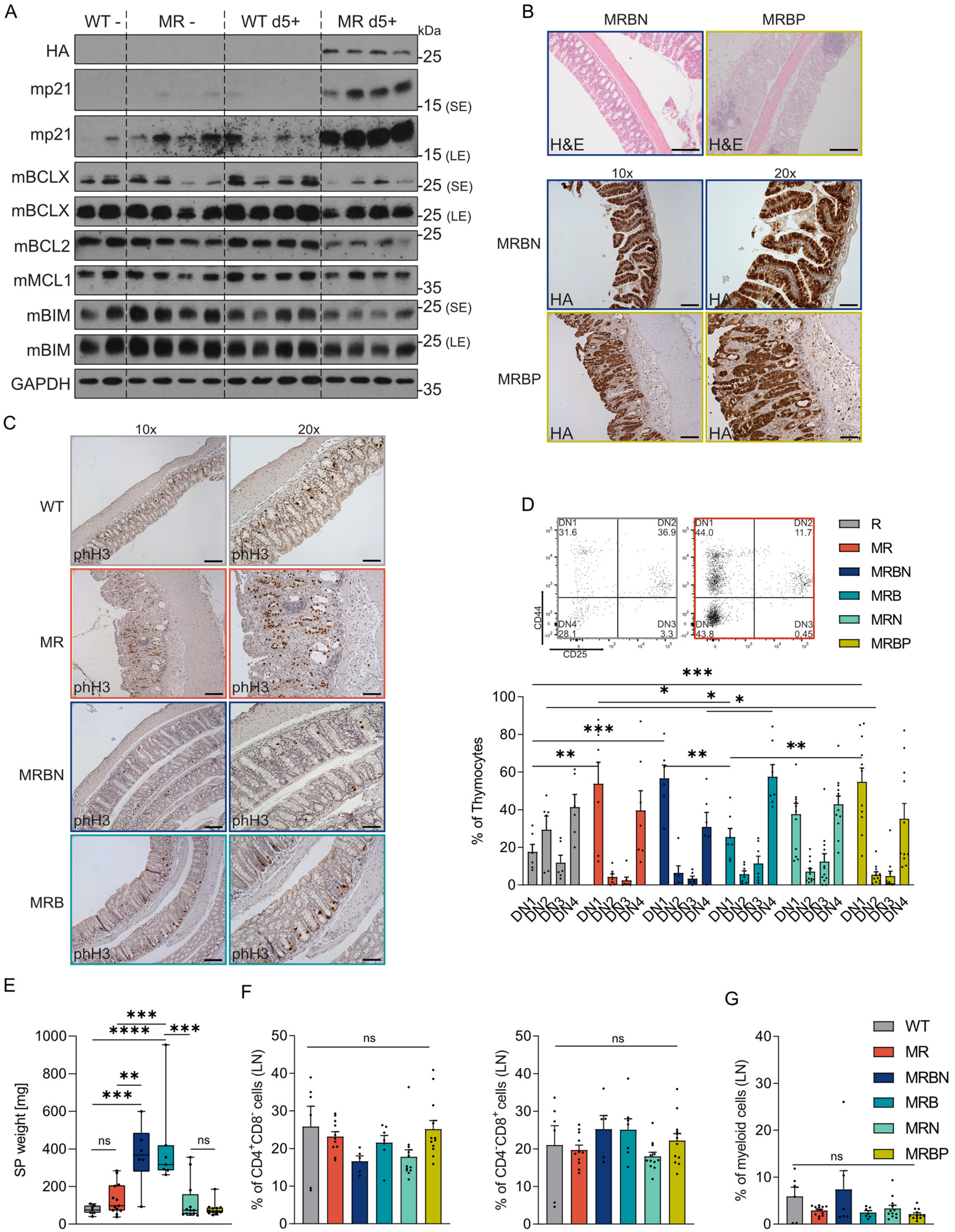
A
WT -
MR -
WT d5+
MR d5+
kDa
HA
mp21
mp21
mBCLX
mBCLX
mBCL2
mMCL1
mBIM
mBIM
GAPDH
25
15 (SE)
15 (LE)
25 (SE)
25 (LE)
25
35
25 (SE)
25 (LE)
35
B
MRBN
MRBP
H&E
10x
20x
HA
C
WT
MR
MRBN
MRB
phH3
D
R
MR
MRBN
MRB
MRN
MRBP
CD44
CD25
% of Thymocytes
DN1
DN2
DN3
DN4
E
SP weight [mg]
ns
F
% of CD4+CD8- cells (LN)
% of CD4-CD8+ cells (LN)
G
% of myeloid cells (LN)
WT
MR
MRBN
MRB
MRN
MRBP

Figure EV4. MAD2 overexpression causes gastrointestinal syndrome.

(**A**) Colonic extracts of mice of the indicated genotypes were subjected to immunoblotting using the indicated antibodies. (**B**) H&E and HA staining of paraffin-embedded sections from Swiss rolls of the small intestine (SI) of MRBN and MRBP animals placed on Dox food; scale bar for H&E staining 200 μM, scale bar for HA staining 1 mm. (**C**) Swiss roll sections of the colon of mice with indicated genotypes were analyzed for the presence of mitotic cells ($phH3^+$); scale bar 1 mm. (**D**) Gating strategy used in flow cytometric analyses of $CD4^-CD8^-$double-negative (DN) thymocytes stained with antibodies for cell surface markers discriminating stages DN1 ($CD44^+CD24^-$), DN2 ($CD44^+CD24^+$), DN3 ($CD44^-CD24^+$) and DN4 ($CD44^-CD24^-$). (**E**) Spleen (SP) weight from healthy and terminally sick animals. (**F**, **G**) Lymph nodes (LN) stained for helper ($CD4^+$) or cytotoxic ($CD8^+$) T cells and myeloid cells ($Gr1^{+/-}CD11b^+$). WT ($n = 6$–7), MR ($n = 7$–13), MRBN ($n = 5$–6), MRB ($n = 7$), MRN ($n = 12$) and MRBP ($n = 11$–12). Data information: (**D**, **F**, **G**) Data shown as mean ± SEM. (**E**) Data shown as Min to Max with median and IQR of: WT 76.5, MR 152, MRBN 386, MRB 252.5, MRN 68, MRBP 29. (**D**) Two-way ANOVA, Tukey's multiple comparisons. (**E**–**G**) One-way ANOVA, Tukey's multiple comparisons. *$P \leq 0.05$, **$P \leq 0.01$, ***$P \leq 0.001$, ****$P \leq 0.0001$.

 © The Author(s)

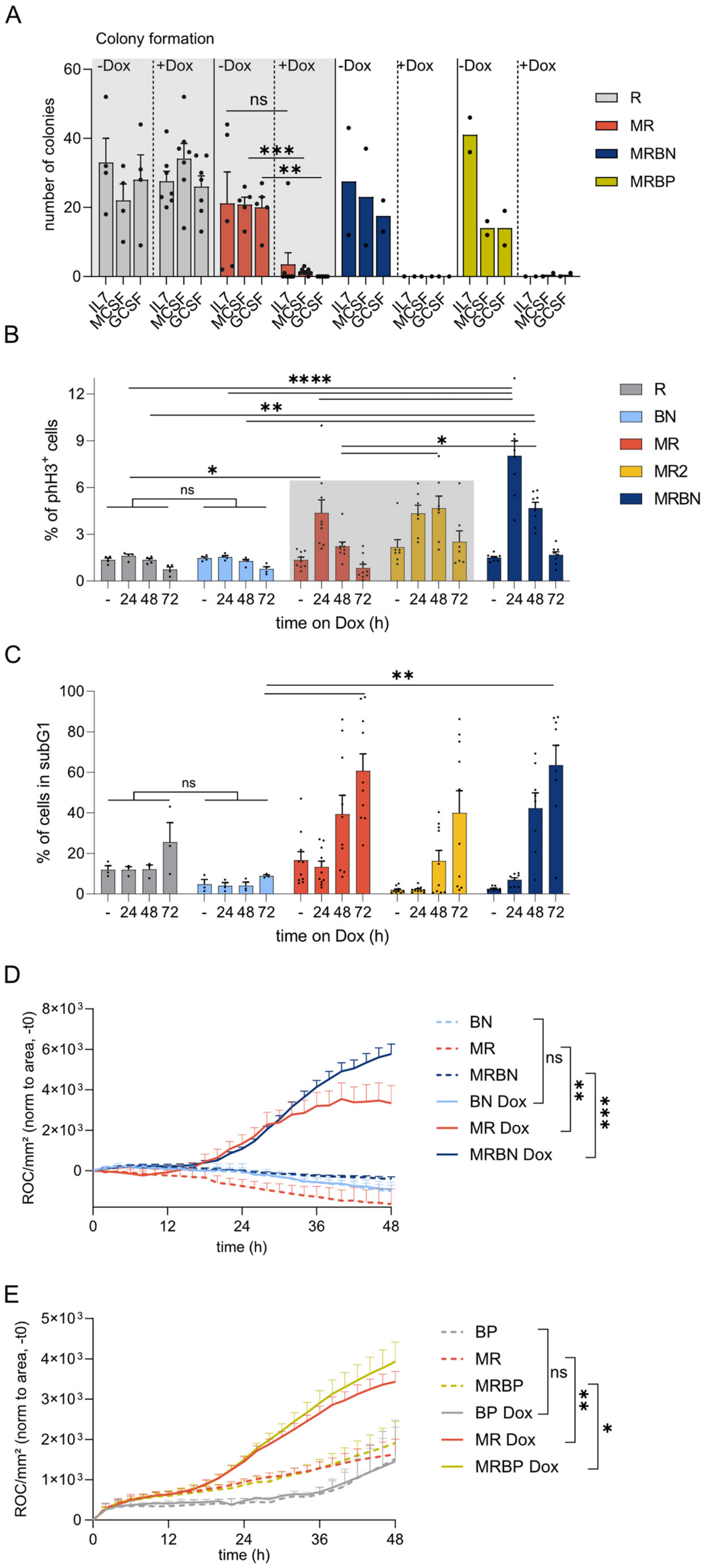
A
Colony formation
number of colonies
-Dox
+Dox
ns
***
**
IL7
MCSF
GCSF
R
MR
MRBN
MRBP
B
% of phH3+ cells
****
**
*
ns
time on Dox (h)
R
BN
MR
MR2
MRBN
C
% of cells in subG1
**
ns
time on Dox (h)
D
ROC/mm² (norm to area, -t0)
time (h)
BN
MR
MRBN
BN Dox
MR Dox
MRBN Dox
ns
**
***
E
ROC/mm² (norm to area, -t0)
time (h)
BP
MR
MRBP
BP Dox
MR Dox
MRBP Dox
ns
**
*

© The Author(s)

Figure EV5. BH3-only protein loss fails to rescue blood cells from mitotic cell death.

(**A**) Colony formation potential of R, MR, MRBN and MRBP transgenic bone marrow was assessed from individual mice in methylcellulose assays in the presence (+) or absence (-) of Doxycycline. (Data points of R and MR, ± Dox of Fig. 3A are shown again for reference). R+Dox ($n = 7$), MR+Dox ($n = 8$), R-Dox ($n = 4$), MR-Dox ($n = 5$). MRBN ($n = 2$), MRBP ($n = 2$). (**B**, **C**) HoxB8-PF cells established from Mad2 transgenic animals were fixed and analyzed by flow cytometry for the presence of mitotic (**B**, phH3^{+}) and dead (**C**, sub-G1) cells (Data points of MR and MR2 cells of Fig. 1C are shown again for reference). R ($n = 2$ biological replicates, 1 measured three times), MR ($n = 9$; 6 biological and 1–2 technical replicates), MR2 ($n = 7$; 3 biological and 1–3 technical replicates), BN ($n = 4$ biological replicates), MRBN ($n = 8$, 6 biological and 1–3 technical replicates); mean ± SEM. (**D**) HoxB8-PF cells were incubated with and without Doxycycline (1 µg/ml). Cell death was recorded over time by Incucyte live-cell microscopy and PI uptake; pictures were taken every 2 h. BN ($n = 5$; 1 biological and 5 technical replicates), MR ($n = 5$; 1 biological and 5 technical replicates), MRBN ($n = 5$; 1 biological and 5 technical replicates). (**E**) HoxB8-PN cells were incubated with and without Doxycycline (1 µg/ml). Cell death was recorded over time by Incucyte live-cell microscopy and PI uptake; pictures were taken every 2 h. BP ($n = 2$, 1 biological and 2 technical replicates), MR ($n = 6$, 4 biological and 2 technical replicates), MRBP ($n = 4$, 2 biological and 2 technical replicates). Data information: (**A–E**) Data shown as mean ± SEM. (**A**) Unpaired *t* test, Welchs correction. _*(**D**, **E**) Unpaired *t* test, Welchs correction for 48 h time point. *_ (**B**, **C**) Two-way ANOVA, Tukey's multiple comparisons. *$P \leq 0.05$, **$P \leq 0.01$, ***$P \leq 0.001$, ****$P \leq 0.0001$.

 © The Author(s)
